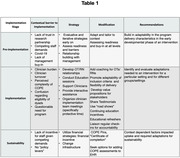# Adapting Implementation Strategies to Context: Moving an Evidence‐Based Dementia Care Program into PACE

**DOI:** 10.1002/alz.092495

**Published:** 2025-01-09

**Authors:** Nancy A Hodgson

**Affiliations:** ^1^ University of Pennsylvania, Philadelphia, PA USA

## Abstract

**Background:**

Systematic approaches are essential for adapting and tailoring implementation strategies to specific dementia care settings if we are to move evidence‐based practice into ‘real world’ care that reaches most persons living with dementia. Care of Persons with Dementia in their Environments (COPE) is an evidence‐based dementia care program that provides families with skills to maximize functional abilities and quality of life of persons with dementia and reduce difficulties managing day‐to‐day care challenges for family caregivers. The COPE in PACE study was a national non‐inferiority trial that involved the implementation of COPE in Programs of All‐Inclusive Care of the Elderly (PACE)‐ which provide support for nursing home eligible Medicaid and Medicare enrollees (NCT04165213). Most PACE enrollees have cognitive impairment or dementia yet not one of the 200+ proven caregiver supportive programs have been integrated in this setting.

**Method:**

Multiple data collection methods including observations, surveys, interviews, and focus groups were used to identify implementation barriers and the resulting program adaptations. Transcripts, templated notes, and field notes gleaned from these sources were cataloged according to the framework for reporting adaptations and modifications to evidence‐based interventions (FRAME‐IS).

**Result:**

The leading barriers to implementation were lack of PACE staff protected time, high staff turnover, low ‘readiness’ in dementia caregivers and lack of dementia‐specific knowledge. Program adaptation strategies were made to improve the feasibility, reach/engagement and fit at the site level and are summarized in Table 1. Different implementation approaches were required at each PACE site and were driven by context dependent factors.

**Conclusion:**

It is important not to underestimate the context dependent factors that may impact uptake of evidence‐ based programs. The array of data collection methods allowed for a greater understanding of the barriers to implementation and reasons for adaptation and can provide guidance for future researchers. The FRAME‐ IS model was useful for documenting adaptations made while implementing COPE in PACE and helped illustrate how adaptations clustered into unique prototypes. Our findings demonstrate how adaptability in delivery characteristics is essential for the translation of a dementia care program into real world practice and provide an example methodology for tracking these changes.